# Comparative analysis of follicle morphology and oocyte diameter in four mammalian species (mouse, hamster, pig, and human)

**DOI:** 10.1186/1743-1050-3-2

**Published:** 2006-03-01

**Authors:** Jeanine Griffin, Benjamin R Emery, Ivan Huang, C Matthew Peterson, Douglas T Carrell

**Affiliations:** 1 Andrology and IVF Laboratories, Division of Urology, Department of Surgery, University of Utah School of Medicine, Salt Lake City, Utah 84108, USA; 2 Department of Physiology, University of Utah School of Medicine, Salt Lake City, Utah 84108, USA; 3 Division of Reproductive Endocrinology, Department of Obstetrics and Gynecology, University of Utah School of Medicine, Salt Lake City, Utah 84132, USA

## Abstract

**Background:**

Laboratory animals are commonly used for evaluating the physiological properties of the mammalian ovarian follicle and the enclosed oocyte. The use of different species to determine the morphological relationship between the follicle and oocyte has led to a recognizable pattern of follicular stages, but differences in follicle size, oocyte diameter and granulosa cell proliferation are not consistent across the different species. In an effort to better understand how these differences are expressed across multiple species, this investigation evaluates oocyte and follicle diameters and granulosa cell proliferation in the mouse, hamster, pig, and human.

**Methods:**

Histological sections of ovaries from the mouse, hamster, pig, and human were used to calculate the diameter of the oocyte and follicle and the number of granulosa cells present at pre-determined stages of follicular development. A statistical analysis of these data was performed to determine the relationship of follicular growth and development within and between the species tested.

**Results:**

These data have revealed that the relationships of the features listed are tightly regulated within each species, but they vary between the species studied.

**Conclusion:**

This information may be useful for comparative studies conducted in different animal models and the human.

## Background

In an effort to understand follicular growth and oocyte development in the human, many animal models of folliculogenesis are in use [1-6]. Each of these models has specific similarities to the human and where one model may be inadequate, another may provide the appropriate characteristics for experimentation. A major obstacle in the interpretation of data from different species in relation to the human lies in understanding the similarities and variances between the investigational systems and the human.

At first glance, the follicular stages of maturation seem to be morphologically well defined across species. In fact, a follicle from any mammalian model can be generally categorized as primordial, primary, or secondary based on the presence and number of cuboidal granulosa cell layers [5,7]. Secondary follicles can then be further subdivided into various stages based on the size and presence of antral fluid. These stages are initially defined as preantral (prior to the accumulation of antral fluid) or antral (after the accumulation of antral fluid). Antral stages are further clarified into stages of incipient antral (from the first signs of fluid accumulation) to later stages of early antral and Graafian stages based on the size of the follicle and amount of follicular fluid [5,8]. However, important variables such as oocyte diameter and the number of supporting granulosa cells are not evaluated in this universally applied classification system [2,9].

Until now, there has not been a study comparing multiple species or indicating the morphological differences that are present in a follicle and its enclosed oocyte at given stages in a single study. This study was therefore designed to simultaneously evaluate the variances in the oocyte and follicle diameter and granulosa cell proliferation within the mouse, hamster, pig, and human at all stages of maturation.

## Methods

### Ovarian tissue collection

The appropriate ethics committee approval was obtained for the use of animal and human ovarian tissue in this study. Female B6D2/F1 hybrid mice and Golden Syrian hamsters were obtained at 3–4 weeks old (Charles River Laboratories, Wilmington, MA) and housed until used for experimentation at six to eight weeks of age. Ovarian tissue was obtained at necropsy immediately after euthanasia and washed twice in 0.01 M PBS. Pig ovaries were collected from pre-pubertal gilts at a local abattoir. These ovaries were transported in 0.01 M PBS containing 3% bovine serum albumin (BSA) (Sigma Chemical, La Jolla, CA) to the site of processing within one hour of removal. Human ovaries were collected from women, 23 to 45 years of age, undergoing oophorectomy for non-neoplastic indications. Human ovarian tissue was removed by the operating surgeon and delivered to the pathology department where a section of ovarian cortex was obtained for study. The ovarian cortex arrived at the site of processing in L-15 Leibovitz media (Invitrogen, Carlsbad, CA) containing 3% BSA within one hour of oopherectomy. All tissues were then transferred to 10% formalin (Sigma Chemical, La Jolla, CA) in 0.01 M PBS for histological processing and evaluation.

### Histological processing and follicle identification

The formalin-preserved tissues from all species were sent to a university core laboratory for routine processing in an automated tissue processor and embedded in paraffin. Five to ten micron serial sections obtained from a rotary microtome were mounted onto plain glass slides and routinely stained with haematoxylin and eosin for light microscopy evaluation.

Each tissue section was evaluated for the presence of oocytic follicles using a Nikon E300 microscope equipped with four, ten, twenty, forty, and sixty times magnification Plan objectives (Nikon, Japan). The microscope was also fitted with a Cool Pix digital camera (Photometrics, Tucson, AZ) at a trinocular mount and interfaced to a Macintosh G4 (Apple Computer, Cupertino, CA) running the image capture software RS Image (Photometrics, Tucson, AZ).

**Figure 1:**
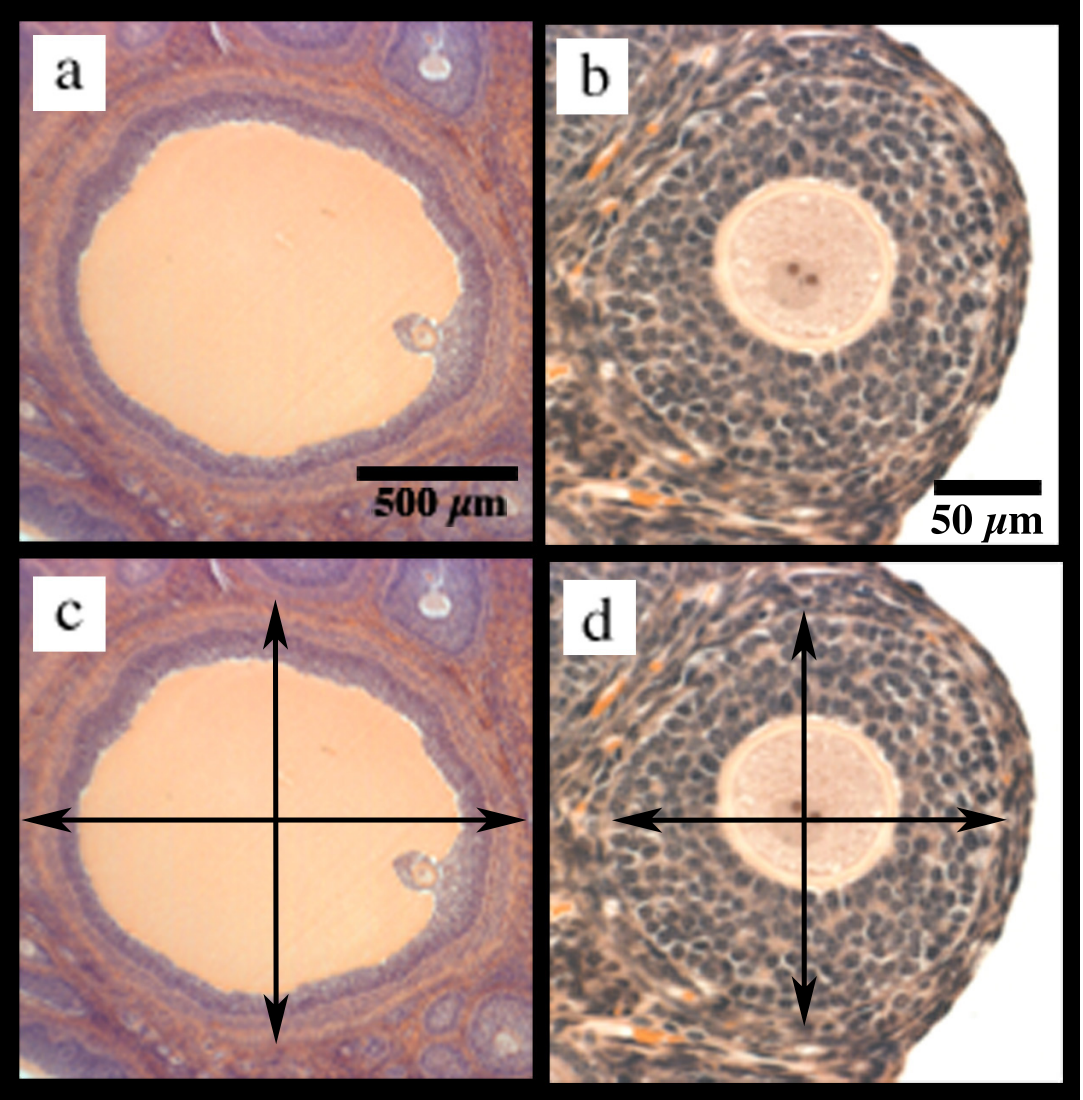
**Follicle Selection**. Depiction of a representative Graafian follicle (pig) at the largest cross-sectional diameter (a), and a representative preantral follicle (hamster) at the midsection (b) and examples of diameter measurements with and without inclusion of the thecal layer, (c,d) respectively.

The identification of follicles within the serial sections was based on strict criteria. Follicles were first assessed to determine if an antral cavity had formed within the follicle. This was carried out by reviewing each appearance of the follicle, across serial sections, for antrum formation or an area within the structure containing a space void of granulosa cells. If an antral cavity was recognized, the serial section of the follicle showing the largest cross-sectional area was then used for further evaluation (Fig. 1a). When the initial assessment of the follicle did not indicate an antral cavity, the section where the oocyte nucleus was visible in the follicle was used for further evaluation (Fig. 1b). Based on the criteria of Gougeon [7] and Knigee and Laetham [8], all follicles were concurrently assessed for morphological signs of atresia and excluded from the study when identified. Furthermore, markedly distorted follicles, likely damaged during tissue preparation, were also excluded from the study. The captured images of all follicles meeting the selection criteria were saved as tiff formatted images and transferred to Image J (NIH, Bethesda, MD), an open source application for data analysis, also running on a Macintosh G4, for further evaluation.

### Follicle and oocyte measurements

The accurate calculation of diameters was ensured by using the integrated measuring tools in the Image J software after calibration with a stage micrometer (Gurley Precision Instruments, Troy, NY). Additionally, when measuring diameters, two measurements were taken. The second measurement originated at a right angle from the midpoint of the first measurement (Fig 1c). The two measurements were averaged and expressed as the diameter of the structure.

Data was collected in this manner to determine the diameters of the follicle, antral cavity, and the oocyte. Follicular diameters were measured from the outer wall of the thecal layer, when present, or from the outer layer of granulosa cells when the thecal layer was absent. The formation of the thecal layer always occurred in the preantral follicle and is present in all later stages. Additionally, the follicles containing a thecal layer were measured across the follicle from inside the thecal layer to aid in calculation of the area of the follicle occupied by granulosa cells (Fig. 1d). The measurements for the antral cavity were from the inner layer of the granulosa cells to the outer layer of the cumulus cells surrounding the oocyte [10]. The oocyte was measured including the zona pellucida, when present. The formation of the zona pellucida always occurred during the preantral follicle stage and is present in all later stages.

**Figure 2:**
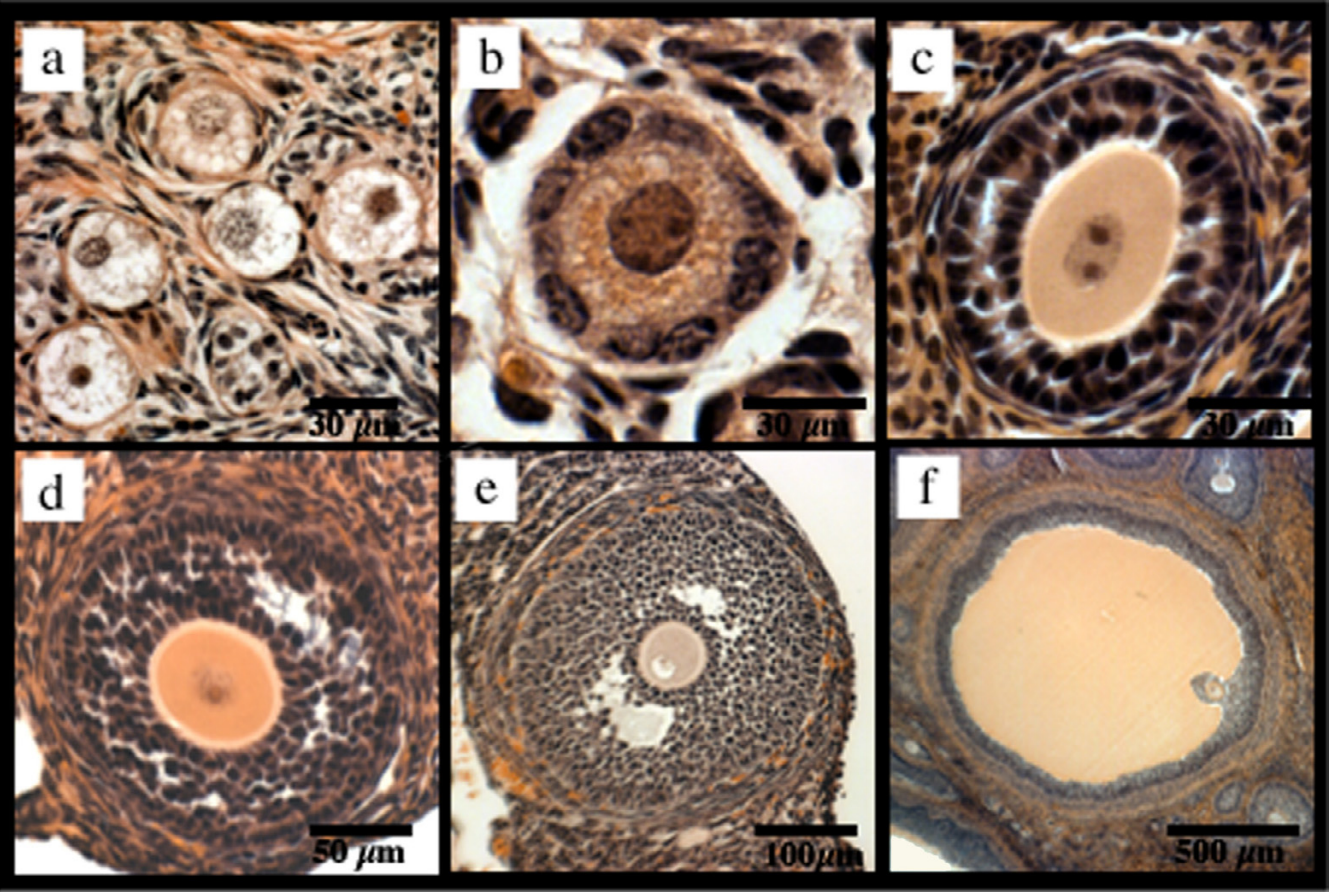
**Follicular Stages of Maturation**. Depiction of representative primordial (pig) (a), primary (pig) (b), preantral (hamster) (c), incipient antral (mouse) (d), small antral (hamster) (e) and Graafian (pig) (f) follicles.

From the above measurements and morphological observations, all follicles across all species were staged (Fig. 2) [1,2,8]. Briefly, oocytes without a zona pellucida and up to one layer of flattened granulosa progenitor cells were classified as primordial follicles. Primary follicles were classified as oocytes surrounded by one layer of cuboidal granulosa cells. Oocytes with two or more layers of granulosa cells but no visible space between granulosa cells were identified as preantral follicles. Antral follicles, those containing any antral cavity, were further divided into categories of incipient and small antral and Graafian follicles. The incipient follicles, which indicate the beginning of antral formation, were identified by the presence of visible space between granulosa cells. Small antral follicles were identified by the presence of a segmented cavity with two or more compartments, while the Graafian follicles contained one large continuous antral cavity.

### Data calculations and statistics

The cross-sectional area of each follicle was calculated according to the equation for the area of a circle, area = πr^2 ^where r, the radius, is equal to half the calculated diameter of the follicle. The number of granulosa cells present in the follicle was derived from manual counting of each cell in the cross-section of the follicle from printed hardcopies of the digital image. Statistical analysis and data comparison were performed using STATA 7.0 (Stata Corporation, College Station, Texas) and Excel 2004 (Microsoft Corporation, Seattle, WA).

## Results

### Follicular diameter

**Figure 3:**
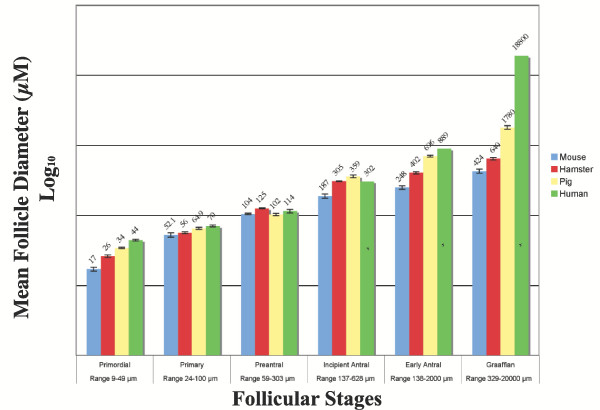
**Follicular Growth in Maturational Stages**. Semi-log bar graph of follicle diameter versus follicular stage, * indicates values from previously published data for reference [11], and therefore do not include error bars. Values are the mean ± standard error.

The measurements obtained for follicle diameter were stratified according to follicular stage classification for all species (Fig. 3). The dataset compiled includes data from this study for follicles from, mouse (n = 104), hamster (n = 273) and pig (n = 284). These follicles were collected from 10 mice, 10 hamster, and 6 pig ovaries. Additionally, human ovarian tissue from 5 biopsy samples was obtained for this study and provided adequate numbers of follicles for analysis up through the preantral stage (n = 126). Thereafter, the appearance of antral follicles that are not atretic in the human become extremely rare and are not likely obtainable using any histological technique [3]. Mean follicle diameter and ranges for the antral groups in the human are therefore listed from previously published data for comparison but not included in the statistical analysis [11].

Interestingly, follicle diameters are significantly different between the four species at the primordial stage, when compared using ANOVA (p < 0.005). Thereafter, follicle sizes converge at the primary and preantral stages only to then see a dramatic disparity with a smaller follicular diameter of the mouse as compared to hamster and pig follicle size at the incipient antrum stage onward (p < 0.001). The pig and hamster diverge from similarity at the early antral stage (p < 0.001).

### Oocyte diameter

**Figure 4:**
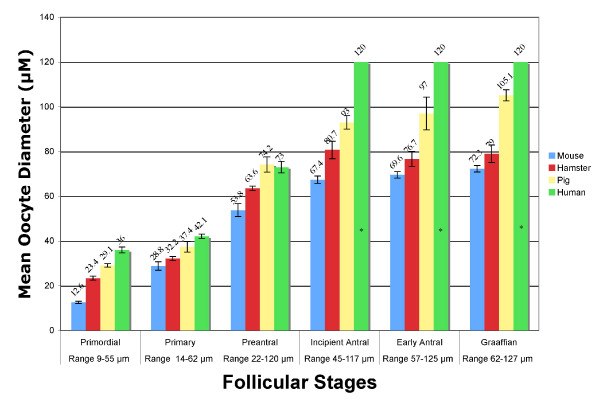
**Oocyte Growth in Maturational Stages**. Bar graph of oocyte diameter versus follicular stage, * indicates values from previously published data for reference [12], and therefore do not include error bars. Values are the mean ± standard error.

The calculated oocyte diameters, for all species, were stratified into the stages of follicular growth (Fig. 4) in the same manner as the follicle diameters from above. The mean diameters of the human oocyte at developmental stages marked from the inception of the antrum on were not obtainable in this study design, as was the case for follicular diameter. For comparison, the mean size of a fully mature human oocyte, which is present from the incipient stage follicle onward [12], is included in figure 4.

When stratified into follicular classes, the statistical comparison of oocyte diameters for mouse, hamster, and pig revealed a difference between species at all stages (p < 0.005). Analysis of the human oocyte diameter revealed a difference from all species at the primordial and primary stages (p < 0.01) but is similar to the pig at the preantral stage of development.

### Granulosa cell count

**Figure 5:**
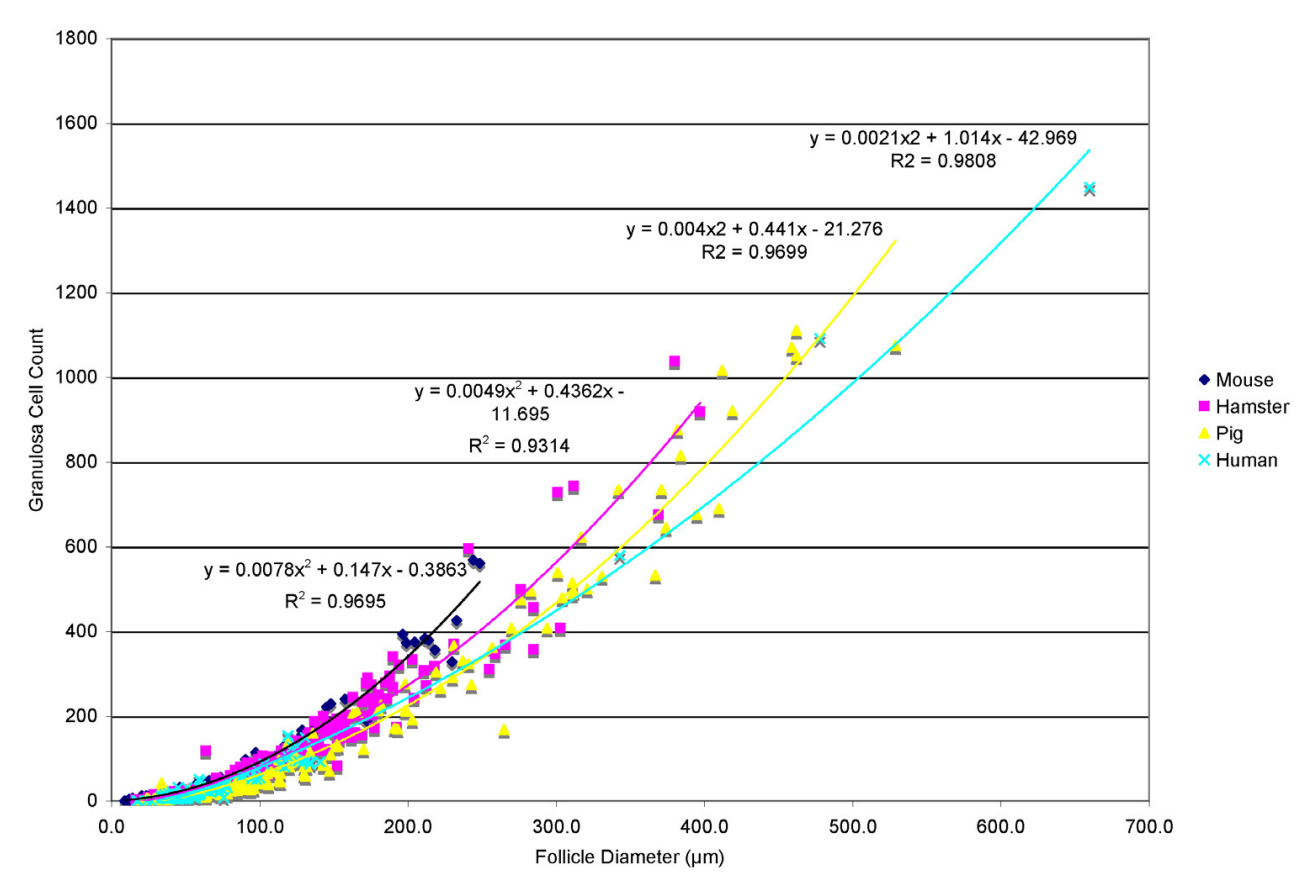
**Granulosa Cell Proliferation**. Regression analysis of granulosa cell count compared to follicle diameter for primary, secondary, and incipient antral follicles in each of the four species.

The number of counted granulosa cells per cross-sectional area of the primordial, primary, secondary, and incipient antral were compared to the follicular diameter of the follicle in each of the four species. The granulosa cells present in Graafian follicles were not evaluated due to the change in doubling times and atresia of granulosa cells within this stage of all species [13]. Small antral follicles were also not included in the analysis due to the increased compaction of granulosa cells from the expanding antral fluid [14]. These factors contributed to make evaluation of cell numbers unreliable by the given method. Regression analysis of granulosa cell count as a function of follicle diameter indicates a quadratic best-fit line for each species (Fig. 5).

## Discussion

### Follicle diameter versus follicular stage

These data indicate that the mean follicle diameters of the species studied have dissimilar growth patterns during follicular development, but do follow a trend of increasing final follicular size within each follicle class in relation to body mass (Fig. 3). The implication from these data is that each species achieves the characteristic morphology of each follicular stage at a defined follicular diameter, as evident by the low standard error of the mean follicle diameter within stages (Fig. 3), but this defined size is not consistent across species.

Furthermore, the growth rate of the follicle as a function of the maturational stage is not consistent within species. In fact, the difference in follicle diameter between stages, within species, becomes progressively greater with each stage. This indicates that the follicle growth from one stage to the next is progressive, but not linear (data not shown). This is consistent with previous publications from individual species showing polynomial growth of the mammalian follicle as a function of time [12,15,16].

### Oocyte diameter versus follicular stage

The comparison of the oocyte diameter is often used as a marker for oocyte maturity or meiotic competence. Whereas this measurement has been used to correlate the stage of follicular development with oocyte maturity within individual species [17,18], these data presented here infer the oocytes of different species reach maturity within the incipient antral stage of development, but the oocyte diameter of each species at maturity is different (Fig. 4). This is validated by previous data showing that in each species studied, the oocyte becomes mature at the inception of the antral fluid accumulation, but the oocyte may continue to grow in diameter to the ovulatory stage [12,18-20].

### Oocyte diameter versus follicle diameter

Another defining morphological feature of the oocyte to follicle relationship is the rate at which the oocyte grows in relation to follicle growth, which can be identified as a type of growth curve. This growth curve is not in direct relation to time, but in relation to the maturation of the follicle and oocyte through the morphological stages of the follicle. The relationship of oocyte to follicular diameter has been previously reported for individual species at the early stages of follicular growth [8,21-23], but this is the first known report comparing this relationship across the four species used in this study (Table 1). The regression equations for the four species are similar, but not identical. In fact, if the oocyte diameters and corresponding follicle diameters are expressed as ratios, an analysis of variance indicates a significant difference in the relationship of the oocyte to follicle diameter between follicular stages and between species (data not shown). Thus, the change in oocyte diameter is not directly proportional to the follicular diameter, even at the early stages of follicular growth.

**Table 1:** Regression Equations for Follicle to Oocyte Diameter

	**Mouse**	**Hamster**	**Pig**	**Human**
**Regression Equation**	y = -0.001x^2 ^+ 0.568x + 2.89	y = -0.001x^2 ^+ 0.569x + 7.02	y = -0.0003x^2 ^+ 0.305x + 20.47	y = -0.0014x^2 ^+ 0.667x + 8.65
**R^2 ^Value**	0.96	0.91	0.81	0.85

### Granulosa cell count versus follicle diameter

Evaluation of the regression analysis of granulosa cell count to follicle diameter reveals that the relationship between granulosa cell proliferation and follicle diameter is tightly regulated within each species and increases in a polynomial fashion (Fig. 3). This correlates with data presented in other mammalian species that have shown the rate of granulosa cell doubling, which is defined as the length of time required for the number of granulosa cells in the midsection of the follicle to double, is much slower in early follicular stages than at the incipient antral stage [3]. Therefore, these data presented are consistent with Hirschfield's observations of follicular growth. In addition, they identify the differences between species as shown (Fig. 3).

## Conclusion

This comparative study is the first to detail the differences observed between experimental models of folliculogenesis. Specifically identified are the morphological variations seen between the mouse, hamster, pig, and human ovarian follicles from histological evaluation. The change in follicle and oocyte diameter in relation to the stage of maturation (Figs. 3, 4), the change in the ratio of follicle to oocyte diameter (Table 1), and the proliferation of follicular cells have all been shown herein to be specific to the species studied and should not be generalized to other models.

This study gives rise to many interesting avenues of thought that may be addressed in future studies. Such studies may include an investigation of why oocytes and follicles are larger in some species than others. Additionally, it would be interesting to identify why the relationship between body mass and follicle size is conserved across species. Is it possible that the increased body size requires a larger fluid volume and oocyte size to facilitate oocyte pick-up in the abdomen for delivery of the oocyte to the fallopian tube for fertilization? These issues may be resolved by further studies manipulating the in vivo system of these species.

## Competing interests

The author(s) declare that they have no competing interests.

## Authors' contributions

JG performed the data collection and summarization. BRE aided in study design data collection, summarization and statistical analysis and drafted the manuscript. IH contributed by performing statistical analysis. CMP and DTC conceived of the study and directed the research design. All authors read and approved the final manuscript.
